# A Novel Seed-Dressing Formulation Based on an Improved Mutant Strain of *Trichoderma virens*, and Its Field Evaluation

**DOI:** 10.3389/fmicb.2019.01910

**Published:** 2019-08-30

**Authors:** Prasun K. Mukherjee, Sayaji T. Mehetre, P. D. Sherkhane, Gopi Muthukathan, Ananya Ghosh, A. S. Kotasthane, N. Khare, Parshuram Rathod, Kishan Kumar Sharma, Rajib Nath, Anand K. Tewari, Somnath Bhattacharyya, Meenakshi Arya, D. Pathak, A. R. Wasnikar, R. K. S. Tiwari, D. R. Saxena

**Affiliations:** ^1^Nuclear Agriculture and Biotechnology Division, Bhabha Atomic Research Centre, Mumbai, India; ^2^Department of Agronomy, Bidhan Chandra Krishi Viswavidyalaya, Mohanpur, India; ^3^Department of Plant Pathology, Indira Gandhi Krishi Vishwavidyalaya, Raipur, India; ^4^Department of Plant Pathology, G. B. Pant University of Agriculture and Technology, Pantnagar, India; ^5^Department of Plant Pathology, Rani Lakshmi Bai Central Agricultural University, Jhansi, India; ^6^Regional Agricultural Research Station, Assam Agricultural University, Shillongani, India; ^7^Department of Plant Pathology, Jawaharlal Nehru Krishi Vishwa Vidyalaya, Jabalpur, India; ^8^R.A.K. College of Agriculture, Rajmata Vijayaraje Scindia Krishi Vishwa Vidyalaya, Sehore, India

**Keywords:** *Trichoderma*, mutant, tamarind seeds, formulation, chickpea, lentil, farmers’ fields

## Abstract

Using gamma-ray-induced mutagenesis, we have developed a mutant (named G2) of *Trichoderma virens* that produced two- to three-fold excesses of secondary metabolites, including viridin, viridiol, and some yet-to-be identified compounds. Consequently, this mutant had improved antibiosis against the oomycete test pathogen *Pythium aphanidermatum*. A transcriptome analysis of the mutant vis-à-vis the wild-type strain showed upregulation of several secondary-metabolism-related genes. In addition, many genes predicted to be involved in mycoparasitism and plant interactions were also upregulated. We used tamarind seeds as a mass multiplication medium in solid-state fermentation and, using talcum powder as a carrier, developed a novel seed dressing formulation. A comparative evaluation of the wild type and the mutant in greenhouse under high disease pressure (using the test pathogen *Sclerotium rolfsii*) revealed superiority of the mutant over wild type in protecting chickpea (*Cicer arietinum*) seeds and seedlings from infection. We then undertook extensive field evaluation (replicated micro-plot trials, on-farm demonstration trials, and large-scale trials in farmers’ fields) of our mutant-based formulation (named TrichoBARC) for management of collar rot (*S. rolfsii*) in chickpea and lentil (*Lens culinaris*) over multiple locations in India. In certain experiments, other available formulations were included for comparison. This formulation consistently, over multiple locations and years, improved seed germination, reduced seedling mortality, and improved plant growth and yield. We also noticed growth promotion, improved pod bearing, and early flowering (7–10 days) in TrichoBARC-treated chickpea and lentil plants under field conditions. In toxicological studies in animal models, this formulation exhibited no toxicity to mammals, birds, or fish.

## Introduction

*Trichoderma* spp. are among the most widely used bioagents in today’s agriculture throughout the world (Mukherjee et al., 2013). The popularity of these fungi stems from their ability to kill other fungi (mycoparasitism), produce several hundred secondary metabolites (some are antimicrobial), induce local and systemic resistance in plants against invading pests and pathogens, improve nutrient (especially nitrogen) use efficiency, promote plant growth, and impart tolerance to abiotic stresses (Lorito et al., 2010). Even though there are more than 200 well-defined species reported in literature, the formulations that are in use are based on only a handful of species like *T. harzianum*, *T. afroharzianum*, *T. viride*, *T. asperellum*, *T. koningiopsis*, and *T. virens* (Atanasova et al., 2013; Mukherjee et al., 2013). The main constraint in using these bioagents has been their inconsistent performance under field conditions, compared to their chemical counterparts that are not much influenced by environmental factors (Zaidi and Singh, 2013). There have been earlier reports to develop novel *Trichoderma* strains using mutagenesis (Papavizas et al., 1982; Ahmad and Baker, 1988; Mukherjee et al., 1997, 1999; Szekeres et al., 2007; Olejníková et al., 2010). However, to the best of our knowledge, these have not been formulated, field-tested, and developed as commercial products. Among the formulations that are commercially available and are successful is one based on a protoplast fusant strain of *T. afroharzianum* (Shoresh et al., 2010; Chaverri et al., 2015; Harman and Uphoff, 2019). We report here improvement of a strain of *Trichoderma virens* that has been widely studied for biocontrol properties (Mukhopadhyay et al., 1992; Mukherjee et al., 2013; Sherkhane et al., 2017), using gamma-ray-induced mutagenesis. In addition to strain improvement, we also report here a novel mass multiplication protocol and a formulation strategy for *Trichoderma*, and report on the non-toxic nature of the *T. virens* mutant formulation in mammals, birds, and fish.

## Materials and Methods

### Strains and Culture Conditions

*Trichoderma virens* IMI 304061 and the plant pathogens *Pythium aphanidermatum* and *Sclerotium rolfsii* were taken from our previous studies (Mukherjee et al., 2007). Fungal cultures were routinely grown in potato dextrose medium and stored in −80°C as glycerol stock for maintaining genetic purity.

### Mutagenesis of *T. virens* and Isolation of a Secondary Metabolite Overproducing Mutant

Sporulated culture (grown in potato dextrose agar slants) of wild-type strain of *T. virens* was irradiated with gamma ray at 1,250 Gy, as described previously (Mukherjee and Mukhopadhyay, 1993). The spores were harvested in sterile distilled water and dilution-plated on PDA amended with rose bengal (100 mg/L) to restrict colony growth. Morphologically different colonies were transferred to fresh PDA plates. One colony having brown color conidia and secreting dark pigments in the medium was purified by repeated single-spore isolation and selected for further studies. Stability of the mutant was tested for 20 generations by repeated subculturing. For antibiosis assay, the strains were grown in PDB for 3 days with continuous shaking, and the filtrate was harvested, passed through 0.22-μm syringe filters, and added to PDA at 3% (v/v). Control was amended with 3% water. The indicator plant pathogen *P. aphanidermatum* was inoculated centrally on filtrate-amended plates and observation was recorded on the growth after 24 h of incubation. The ability of wild type and G2 to overgrow colonies of the plant pathogen *S. rolfsii* was assessed using confrontation assay (Mukherjee et al., 2013). The ability of the strains to colonize and kill sclerotia of *S. rolfsii* was assessed by spreading conidial suspension (100 μl of 10^6^/ml) on 1% water agar plates and then seeding the sclerotia on it. Parasitization of the sclerotia was recorded daily and sclerotial viability was determined by plating on PDA amended with 10 mg/L benomyl (to selectively inhibit the growth of *T. virens*). For HPLC, the filtrates were extracted with ethyl acetate, concentrated (10-fold) using nitrogen flush, and subjected to HPLC analysis as described earlier (Mukherjee et al., 2006).

### Transcriptome Analysis

For RNA extraction, *T. virens* WT and G2 were grown on PDA plates lined with a dialysis membrane (MWCO 12,000–14,000) for 3 days and the tissue (containing both mycelia and conidia) were harvested by scraping with a sterile spatula, ground in liquid nitrogen, and RNA extracted with TriReagent (MRC). A paired-end library was prepared, and *de novo* transcriptome sequencing and assembly were performed on Illumina HiSeq 2500 platform at M/S Scigenom, Cochin, Kerala, India. Adaptor trimming, quality filtering, and end trimming were performed and the cleaned reads were assembled using Trinity software with default settings. All assembled transcripts were found to be of length of more than 200 bp. The trimmed reads were aligned to the assembled transcriptome using the Bowtie2 program. Of all filtered reads, about 95% of reads from each sample were properly aligned back to the assembled transcriptome. Differential gene expression analysis was performed using DESeq program. The assembled transcript was annotated using an in-house pipeline (CANoPI – Contig AnnotatorPipeline) at M/S Scigenome Labs., Cochin, Kerala, India, for *de novo* transcriptome assembly using the following steps: Comparison with NCBI database using BLASTX program, organism annotation, gene and protein annotation to the matched transcript, and domain search by using NCBI-CDD search database. Fold change was calculated based on the FPKM values, and genes having log_2_ fold change ≥1.5 was considered for comparison.

### Mass Multiplication, Formulation, and Seed Treatment

For mass multiplication of *T. virens*, we used a novel medium, i.e., tamarind seeds in solid-state fermentation. Tamarind seed is a cheap by-product of tamarind pulp industry and is available locally in all parts of India. The seeds were cut in four pieces, and 100 g was soaked overnight in 180-ml tap water in a 500-ml conical flask. Even though whole tamarind seeds support profuse growth of *T. virens*, cut seeds were used to increase substrate surface area, resulting in higher fungal biomass production. After autoclaving for 15 min at 15 psi, seeds were inoculated with *Trichoderma* and incubated for 7–10 days. The growth was mixed thoroughly with 400 g of autoclaved talcum powder and sieved through a stainless steel sieve (8 mesh). The powder was air dried and kept refrigerated for further use. The formulation was stable for at least 6 months at 4–6°C, without any significant loss of viability. Colony-forming units were counted by dilution plating on rose bengal agar plates. For seed treatment, 5 to 10 g of the formulation was suspended in 25 ml of water to make a slurry, and seeds were treated with this slurry by shaking in a polybag. Seeds were air dried before sowing.

### Greenhouse Assay for Control of *S. rolfsii*

In order to have a comparative assessment of wild type and G2, we checked for the ability to protect chickpea seeds and seedlings from *S. rolfsii*, a serious pathogen of chickpea (and of more than 500 plant species), in a greenhouse pot experiment. Two kilograms of non-sterile soil was taken in plastic pots. For inoculum, *S. rolfsii* was grown in sorghum grains for 10 days. Four grams (determined experimentally to create high disease pressure, i.e., about 100% mortality) of *S. rolfsii* culture was mixed with top 3 cm soil. The pots were covered for 3 days to help the pathogen get established in soil. Twenty treated (wild type or G2 @ 2.5 and 5 g/kg seeds, treated in slurry form as described above) chickpea seeds were sown per pot in pathogen-infested soil or pathogen-free soil, in three replicates. Observation on seed germination was recorded after 3 days and healthy plant stand was recorded after 7 days.

### Replicated Field Trials for Control of Chickpea Collar Rot

#### Experiment at Crop Research Centre, G.B. Pant University of Agriculture and Technology, Pantnagar, Uttarakhand, India

A replicated trial was conducted at Pantnagar in a field naturally infested with root rot and wilt pathogens (*S. rolfsii*, *Rhizoctonia solani*, and *Fusarium oxysporum* f.sp. *ciceris*) in the 2017–2018 crop season. Individual plot size was 3 m × 2 m, and the experiment was laid out in randomized block design (RBD) with three replicates. Several biocontrol formulations of *Trichoderma* isolates/spp. and *Pseudomonas fluorescens* were compared with our formulation. Chickpea seeds were treated with 5 g/kg seeds (TrichoBARC) or 10 g/kg seeds (other bioagents) formulation, and 240 seeds were sown per plot. Moist seeds were treated with dry talcum-based formulations of the bioagents. Seedling and plant mortality was recorded between 30 and 120 days and mature plant wilt 120 days after sowing (DAS).

#### Trials Under All-India Coordinated Research Project on Chickpea at Four Locations

Multi-location replicated field trials were conducted at Raipur (Chhattisgarh State), Jhansi (Uttar Pradesh), Jabalpur (Madhya Pradesh), and Shillongani (Assam) in the 2017–2018 and 2018–2019 crop seasons. Plot size was 4 m × 3 m and the experiments were laid out in RBD with three replicates. Seeds of chickpea were treated with 1% of the *T. virens* G2 formulation grown in tamarind seeds. Three other strains of *Trichoderma* spp. and chemical seed treatment were included for comparison. Observations of seedling emergence, seedling mortality, and yield were recorded.

### On-Farm Demonstration Trials

On-farm demonstration trials for control of chickpea collar rot was conducted in the 2015–2016 and 2016–2017 crop seasons at Indira Gandhi Krishi Vishwavidyalaya, Raipur, Chhattisgarh. In Raipur in 2015–2016, experiment was taken in a plot of 16 m × 7 m. Chickpea seeds treated with 0.5% TrichoBARC formulation was sown and observation on seed yield and biomass production was recorded. In 2016–2017, 63 m × 24 m plots were used to demonstrate the effect of TrichoBARC seed treatment on chickpea yield.

### Trials in Farmers’ Field

In 2017–2018, trials were conducted in the field of 30 lentil farmers in four villages (Jahirapara, Kurumbelia, Bizra, and Panpur) in Nadia district of West Bengal province, India. Experimental plot size was 0.3 acres. Seeds were treated @ 5 g/kg seeds of Moitri variety, and observations on seedling mortality and grain yield were recorded, and compared with non-treated plots. Trials in farmers’ field were repeated the same way in the 2018–2019 crop season.

### Toxicological Studies

A toxicological study with TrichoBARC formulation was conducted at the Department of Toxicology, IIBAT, Padappai, Tamil Nadu, India. The study was performed in accordance with the US-EPA, OPPTS microbial pesticide test guidelines and Committee for Control and Supervision of Experiments on Animals (CPCSEA) Prevention of Cruelty to Animals Act 1960 (formed in 1964 and revived in 1998). Animals were fasted for 3 h before the treatment and 0.3 ml (twice) containing approximately 10^8^ colony-forming units of the formulation was administered in split dose orally to each animal in the treatment groups (rat, mice, and rabbit). Untreated animals were used as controls. Food was withdrawn for 1 h post-administration. The treated and control animals were sacrificed at various points during the study on days 3, 7, 14, and 21. All animals were observed daily for clinical signs, mortality, and morbidity until the day of their scheduled sacrifice. Body weights of individual animals were recorded shortly before the administration, weekly thereafter, at interim and final sacrifice. For toxicity studies in chicken (*Gallus gallus domesticus*), the desired quantity (20 g) of the test item was weighed and made up to 100 ml using distilled water and thereafter administered to the birds by oral intubation to five female chickens (bird nos. 6–10) at the dose level of 2,000 mg/kg body weight. Five control female chickens (bird nos. 1–5) were similarly treated but with distilled water alone. All birds were observed individually for toxicity signs and mortality thrice on day 0 and thereafter daily for 14 days. The body weight of each bird was recorded just prior to administration of dose (0 day) and on days 3, 7, and 14 after administration. Food consumption was measured daily. Water consumption data were collected on days 3, 7, and 14. At the end of the test, all surviving birds were humanely euthanized by carbon dioxide asphyxiation and gross pathology was conducted on all birds from the treatment group and control. Samples of liver, lungs, kidney, brain, spleen, and blood were collected aseptically and homogenized in cold phosphate buffered saline for microbial evaluation from the treated and control birds at the end of the experimental period. Similar studies were conducted in Pigeon (*Columba livia*). A study was also conducted to evaluate the acute toxicity of the formulation to freshwater fish, *Danio rerio*. In a range-finding experiment, groups of seven fish each in two replicates were exposed to control and test material concentrations of 25.0, 50.0, and 100.0 mg/L TrichoBARC formulation corresponding to 0, 3.9 × 10^5^, 7.8 × 10^5^, and 1.56 × 10^6^ CFU/ml, respectively, for 96 h. The fish were observed for mortality and abnormal behavior twice on the day (3 and 6 h) of exposure and, thereafter, at the end of every 24 h throughout the experimental period. Based on the results of the range-finding experiment, the limit test was conducted with control and test material concentration of 100 mg/L in two replicates at 7 fish/replicate for 96 h. The fish were observed for mortality and abnormal behavior as described above. All the data were analyzed statistically.

## Results and Discussion

### G2 Is a Secondary Metabolite Overproducing Mutant

**FIGURE 1:**
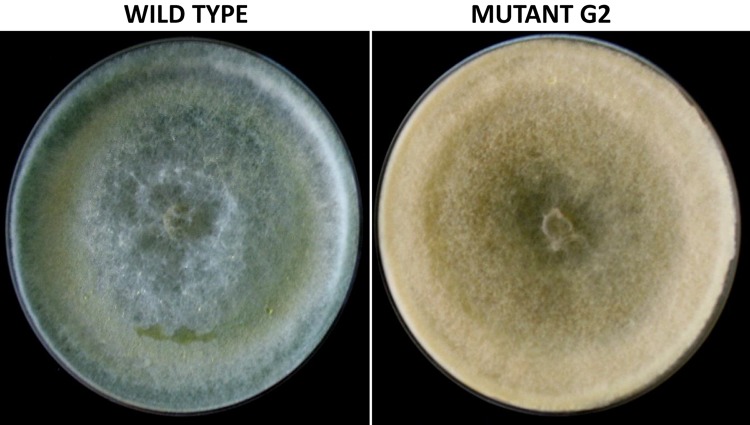
Colony morphology of wild-type and mutant strain of *Trichoderma virens*, after growth on potato dextrose agar for 5 days.

**FIGURE 2:**
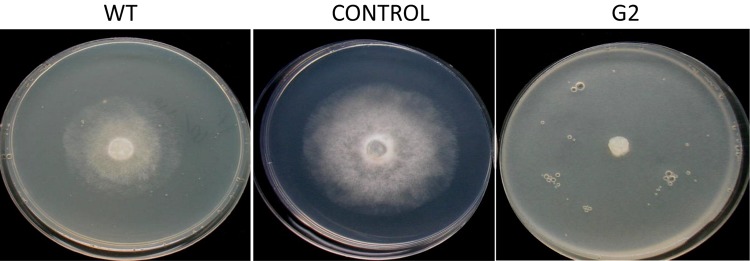
Inhibition of *Pythium aphanidermatum* by culture filtrate of WT and G2.

**FIGURE 3:**
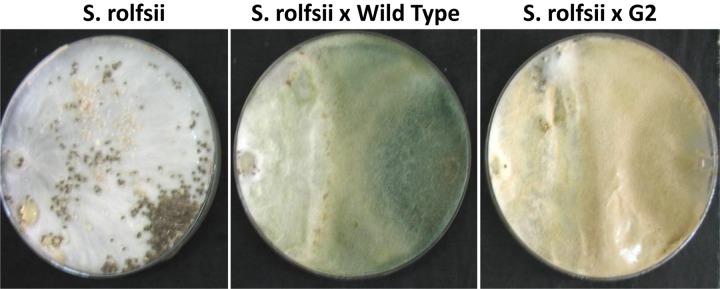
Confrontation assay between *Sclerotium rolfsii* and *Trichoderma virens* mutant/wild-type strain.

**FIGURE 4:**
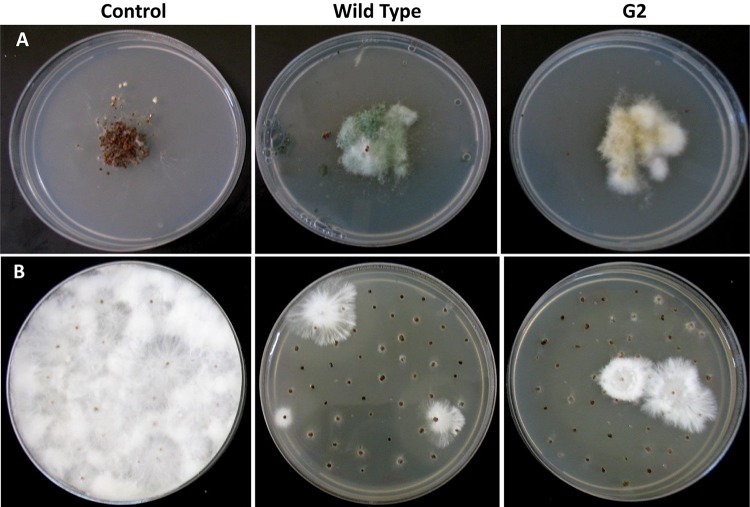
Parasitism of sclerotia of *Sclerotium rolfsii* by wild type and mutant of *Trichoderma virens*. **(A)** Sclerotia colonized after 5 days of co-incubation of *Trichoderma* and sclerotia of *S. rolfsii*. **(B)** Growth of *S. rolfsii* from sclerotia after 3 days of incubation on benomyl-amended PDA medium.

**FIGURE 5:**
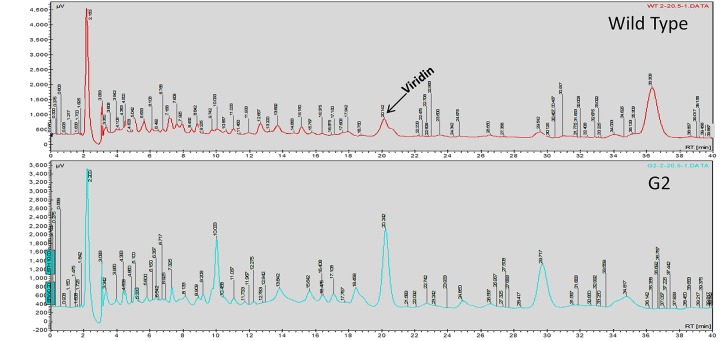
HPLC analysis of culture filtrate of WT and G2.

Exposure to 1,250 Gy of gamma ray followed by selection for varied colony morphology and hyperpigmentation of culture medium (by examining the reverse side of the culture plate) led to the isolation of a stable mutant (designated as G2) that has yellow-brown conidia, dark coloration of the medium, and normal growth and conidiation like the wild-type strain (Figure 1). The mutant, when grown in potato dextrose broth for 3 days in shake culture, produced dark pigment(s) in liquid medium and the filtrate was more inhibitory than the wild type on growth of the test plant pathogen *P. aphanidermatum* (Figure 2). In a confrontation assay, the mutant was as effective as the wild type in overgrowing the plant pathogen *S. rolfsii* (Figure 3). The mutant was also as effective as the wild type in parasitism of the sclerotia of *S. rolfsii* (Figure 4). In HPLC analysis, the mutant produced about 3-fold more of secondary metabolites, including the known metabolites viridin and viridiol, and many yet-to-be identified compounds (Figure 5). These data indicate that the mutant is a hyper-producer of several secondary metabolites, and as effective as the wild type in mycoparasitism. The mutant has been deposited with the Microbial Type Culture Collection, Chandigarh, India (Acc. No. MTCC 11567).

### Several Genes Related to Secondary Metabolism, Mycoparasitism, and Plant Interactions Are Upregulated in G2

**FIGURE 6:**
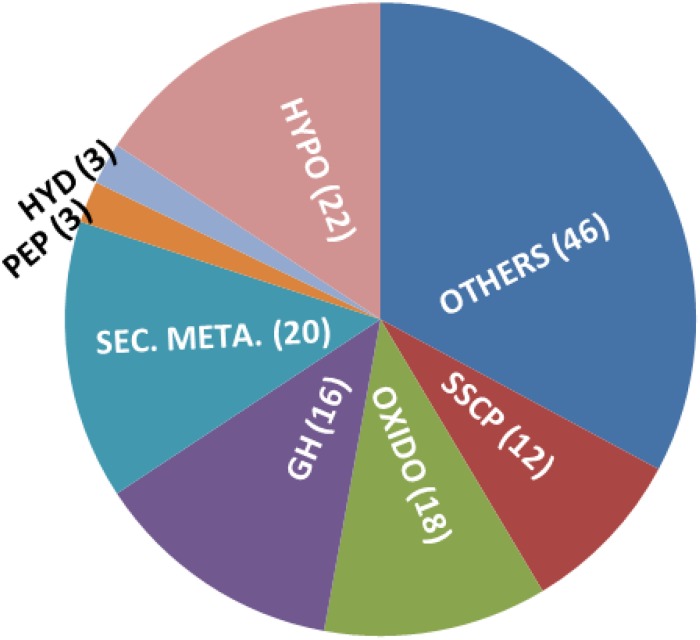
Genes upregulated in *T. virens* mutant G2, over wild type, when grown on PDA for 3 days. Hypo: hypothetical/unknown proteins; SSCP: small, cysteine-rich secreted proteins; OXIDO: oxidoreductases; GH: glycosyl hydrolases; SEC. META.: gene predicted to be involved in secondary metabolism (includes PKS, P450, NRPS-like, OMT-B, and MFS transporters); HYD: hydrophobins; PEP: peptidases.

In order to understand the genomic basis of enhanced secondary metabolite biosynthesis, we did a transcriptome analysis of the mutant vis-à-vis the wild-type strain, grown on potato dextrose agar medium for 3 days. Indeed, several secondary metabolism biosynthesis and transport-related genes like polyketide synthases (6), *O*-methyl transferases (4, including one involved in viridin biosynthesis – Bansal et al., 2018), cytochrome P450s (4), and MFS transporters (4) were upregulated in the mutant, compared to wild type (Figure 6 and Supplementary Table S1). This excludes 16 oxidoreductases that are upregulated in the mutant; oxidoreductases are also known to be part of several secondary metabolism gene clusters and some of these might also be involved in secondary metabolite biosynthesis (Zeilinger et al., 2016). Interestingly, in addition to genes for secondary metabolism, 18 glycosyl hydrolases (GHs) are also upregulated in the mutant. This is surprising because most of the GHs are under catabolite repression in glucose-rich medium (like PDA). Several GHs like chitinases, chitosanases, and beta-glucanases that are upregulated in the mutant are known to be involved in mycoparasitism and biocontrol (Inbar and Chet, 1995; Haran et al., 1996; Carsolio et al., 1999; Djonović et al., 2006b, 2007b). Certain GHs like pectinases are induced during root colonization by *Trichoderma* and are known to be involved in penetration of roots (Morán-Diez et al., 2009, 2015). Small cysteine-rich secreted proteins (SSCPs) are a broadly defined group of proteins that are secreted, small (300 amino acids or less), and have at least four cysteine residues (Mendoza-Mendoza et al., 2018). Many of the proteins involved in plant–microbe interactions (as elicitors and effectors) are SSCPs, for example, Sm1 and Sm2 of *T. virens* as elicitors and four SSCPs as effectors that presumably facilitate root internalization (Djonović et al., 2006a, 2007a; Gaderer et al., 2015; Lamdan et al., 2015; Mendoza-Mendoza et al., 2018). In this study, as many as 12 SSCPs are upregulated in mutant G2 over wild type, even when it was grown on PDA, and not in association with roots. In addition, an ortholog of *hce2*, gene encoding a pathogen effector and a putative necrosis-inducing factor (Ecp2 is a member of this family), is also induced in G2. *Trichoderma* hydrophobins are known to be involved in attachment of hyphae to roots – deletion of *hyd2* resulted in compromising cucumber root colonization by *T. asperellum* (Viterbo and Chet, 2006). *Trichoderma* hydrophobins are also reported to be involved in mycoparasitism, plant growth promotion, and induced resistance (Ruocco et al., 2015; Guzmán-Guzmán et al., 2017; Przylucka et al., 2017; Yu et al., 2019; Zhang et al., 2019). Three hydrophobins are upregulated in G2, compared to wild type. In short, many genes that can have a positive effect on plants are induced in G2 even when grown on a nutrient-rich medium not under induction by its host fungi or plants.

### Tamarind Seeds Support Profuse Growth of *T. virens*

**FIGURE 7:**
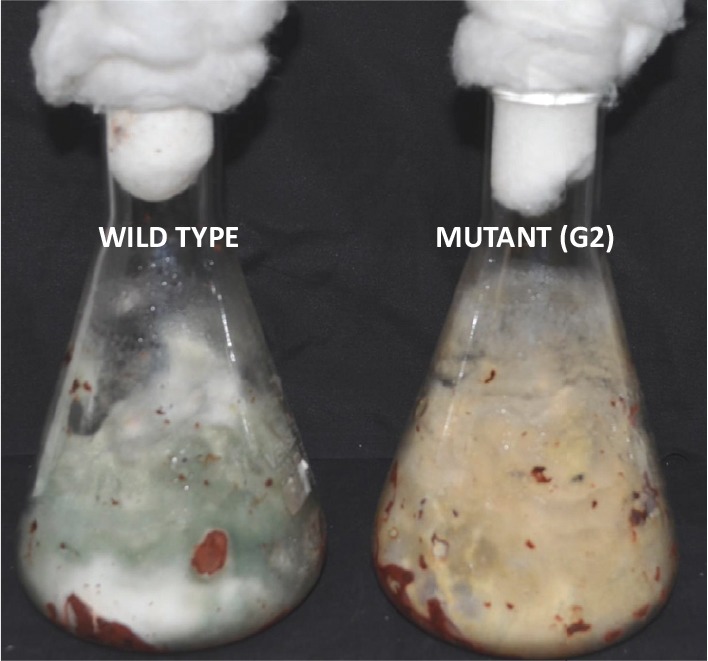
Growth of *Trichoderma virens* wild type and mutant on tamarind seeds, 7 days after inoculation.

**FIGURE 8:**
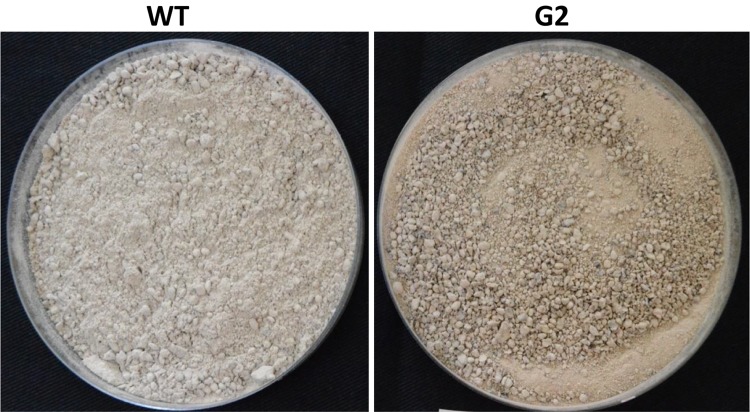
Formulated product of *Trichoderma virens* wild type and mutant.

**FIGURE 9:**
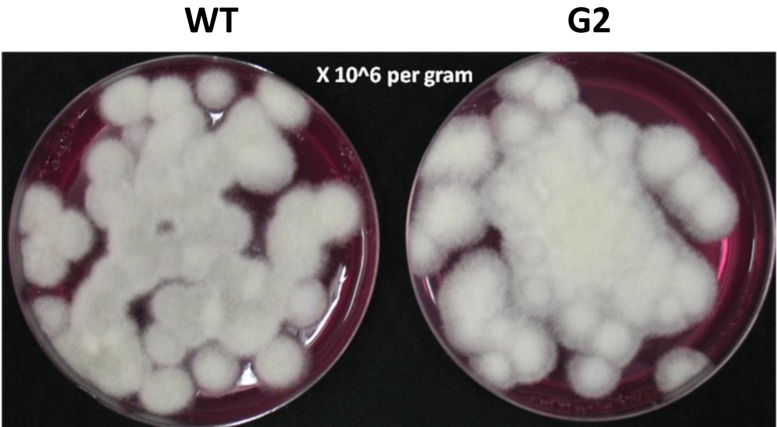
Colony-forming units of *Trichoderma virens* on PDA-rose bengal medium.

Tamarind seeds are a by-product of tamarind pulp industry and are cheap and locally available. In order to replace widely used food grains as a medium for growth of *Trichoderma*, we tested the ability of tamarind seeds to support growth and conidiation of *T. virens* in solid-state fermentation. Broken tamarind seeds supported profuse growth and conidiation of both wild type and the mutant (Figure 7). The final formulation with talcum powder (80%, W/W) appeared darker for the mutant, because of hyperpigmentation (Figure 8). The colony-forming units were determined to be 10^8^/g for both the wild type and the mutant formulations (Figure 9). The formulation was free of any bacterial or mold contamination.

### G2-Based Formulation Is Superior to Wild Type in Greenhouse Assays

**FIGURE 10:**
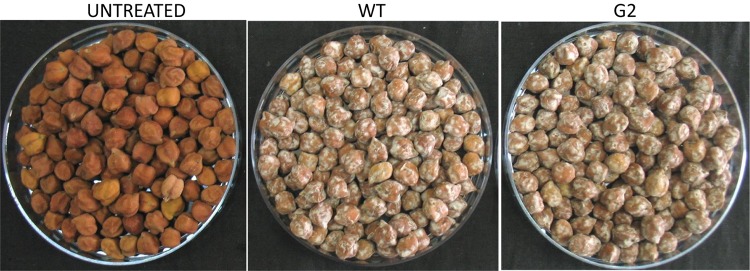
Seeds treated with wild type and G2 formulations.

**FIGURE 11:**
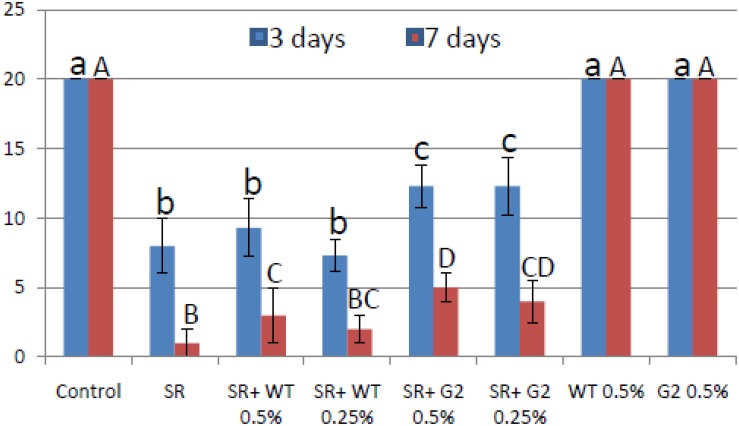
Pot assay for biocontrol of *Sclerotium rolfsii* in chickpea treated with wild type or mutant, in the presence and absence of the pathogen. Treatments with same letters (case-sensitive) are not significantly different (*P* < 0.05).

Most *Trichoderma* formulations available in the market have added amendments like carboxy-methyl cellulose and/or a detergent that acts as a spreader/sticker. Hence, *sensu stricto*, these formulations are not organic. Tamarind seeds have intrinsic sticking properties as it is rich in xylo-glucan (Mishra and Malhotra, 2009). Seeds could be easily coated with this formulation without adding any sticker (Figure 10). When coated chickpea seeds were sown in pot soil heavily infested with the pathogen *S. rolfsii*, the mutant-based formulation showed improved disease control potential compared to wild type (Figure 11). This experiment was repeated several times and the superiority of the mutant to reduce seedling mortality was reproducible. We therefore proceeded to field trials with the mutant-based formulation (named TrichoBARC) at multiple locations over several years.

### The Formulation Is Effective in Reducing Seedling Mortality and Improving Yield of Chickpea Under Field Conditions

In a replicated filed trial at Pantnagar conducted during the 2017–2018 crop season in soil naturally infested with collar rot, root rot, and wilt pathogens, TrichoBARC formulation significantly reduced chickpea plant mortality (11.6% wilted plants, as against 36.9% in control plot) and was superior to other *Trichoderma* or *P. fluorescens*-based formulations (Table 1). In multi-location trials on collar-rot control, TrichoBARC was evaluated and compared with a few other *Trichoderma* strains, fungicides, and combination treatment, at four locations/states of India over 2 years. TrichoBARC treatment of seeds significantly improved seedling emergence, reduced disease incidence, and improved yield in all four locations over both years (Tables 2A–C, 3A–C).

**TABLE 1:** Efficacy of bio-agents against seed and plant mortality of chickpea in field at Crop Research Centre, Pantnagar (2017–2018).

**Treatment**	**Plant stand (30 DAS) ± SE (*N*)**	**Germination (30 DAS) (%)**	**Plant stand (30–120 DAS) ± SE (*N*)**	**Plant mortality (30–120 DAS) (%)**	**Plant stand (120 DAS) ± SE (*N*)**	**Mature plant wilt (120 DAS) (%)**	**Plant mortality including wilted plants (30–120 DAS)(%)**
TCMS-36	157.0 ± 3.2	65.4	144.3 ± 0.9	8.0	130.0 ± 4.6	9.9	17.9
PBAT-3(Ta + Pf)	165.0 ± 2.1	68.7	146.3 ± 4.3	11.3	139.3 ± 3.8	4.7	15.8
Th-14	155.3 ± 4.4	64.7	147.6 ± 4.7	4.9	138.6 ± 6.1	6.0	10.9
Th-17	163.0 ± 4.7	67.9	143.9 ± 2.0	11.7	134.6 ± 4.7	6.4	18.1
Th-39	165.3 ± 7.3	68.8	135.6 ± 5.6	17.9	129.0 ± 7.0	4.8	22.7
Th-19	150.6 ± 3.7	62.7	126.2 ± 4.8	16.2	114.6 ± 6.4	9.1	25.3
Psf-173	155.6 ± 3.2	64.8	128.2 ± 3.4	17.6	121.6 ± 4.3	5.1	22.7
Psf-2	162.6 ± 5.5	67.7	138.3 ± 4.3	14.9	131.0 ± 5.5	5.2	20.1
NBAIR1-Th	167.6 ± 5.5	69.8	142.2 ± 3.3	15.1	129.6 ± 6.5	8.8	23.9
NBAIR2-Ta	153.6 ± 8.0	64.0	124.6 ± 8.2	18.8	112.0 ± 6.5	10.1	28.9
TrichoBARC	158.3 ± 3.8	65.9	147.3 ± 4.1	6.9	140.3 ± 6.4	4.7	11.6
Sanjeevni TV	158.0 ± 5.2	65.8	130.9 ± 1.2	17.1	121.3 ± 5.6	7.3	24.4
Carbendazim	167.6 ± 4.6	69.8	125.2 ± 6.2	25.2	114.6 ± 5.2	8.4	33.6
Control	156.0 ± 3.2	65.0	114.6 + 3.9	26.5	102.6 ± 7.2	10.4	36.9
CD (0.05)	11.1				12.98		
CV (%)	4.1	–	–		130.0	–	–

*Seeds were treated with the following bioagents: TCMS-36: *Trichoderma asperellum;* PBAT-3: *T. asperellum* strain; Th14 + : *Pseudomonas fluorescens* strain Psf 173; Th-14: *T. asperellum*; Th-17: *T. asperellum*; Th-39: *T. harzianum*; Th-19: *T. harzianum*; Psf-173: *P. fluorescens*; Psf-2: *P. fluorescens*; NBAIR1-Th: *T. harzianum*; NBAIR2-Ta: *T. asperellum*; Sanjeevni TV: *T. viride* each @10 g/kg seeds; TrichoBARC: *T. virens* mutant G2 @ 5 g/kg seeds. Other *Trichoderma* and *Pseudomonas* strains were isolated from the Uttarakhand state of India, grown in sorghum grains (*Trichoderma*) or nutrient broth (*Pseudomonas*), and applied as talcum-based non-commercial formulations.*

**TABLE 2A:** Evaluation of new stains of *Trichoderma* along with fungicides for the management of collar rot of chickpea (2017–2018) – Seedling emergence.

**S. No.**	**Treatment Details**	**Raipur ± SE**	**Jhansi ± SE**	**Shillongani ± SE**	**Jabalpur ± SE**	**Mean**
T_1_	*Trichoderma harzianum* T-6 (@10 g/kg seed)	196 ± 28.5	225 ± 5.8	313 ± 19.2	177 ± 9.1	227.75
T_2_	*Trichoderma harzianum* T-28 (@10 g/kg seed)	263 ± 50.5	209 ± 8.7	286 ± 12.0	181 ± 3.1	234.75
T_3_	*Trichoderma viride* T-18 (@10 g/kg seed)	292 ± 8.7	168 ± 19.2	297 ± 9.4	185 ± 8.7	235.50
T_4_	TrichoBARC (@10 g/kg seed)	291 ± 4.7	186 ± 6.6	288 ± 15.8	185 ± 9.3	237.50
T_5_	Propineb (Antracol) @3 g/kg seed	238 ± 66.7	203 ± 4.0	287 ± 12.6	178 ± 4.8	226.50
T_6_	Hexaconazol + Zineb (Avtar) @3 g/kg seed	301 ± 10.8	204 ± 17.1	286 ± 20.0	182 ± 5.8	243.25
T_7_	*Trichoderma harzianum* T-6 + Propineb (@10 g + 1.5 g/kg seed)	182 ± 2.6	273 ± 12.9	309 ± 5.7	182 ± 2.3	236.50
T_8_	Control	181 ± 13.9	140 ± 8.9	277 ± 15.1	175 ± 7.5	193.25
	C.D. at 5%	69.60	25.90	7.91	1.40	
	C.V. (%)	19.54	8.80	13.02−	1.368	

**TABLE 2B:** Evaluation of new stains of *Trichoderma* along with fungicides for the management of collar rot of chickpea (2017–2018) – Disease incidence (%).

**S. No.**	**Treatment Details**	**Raipur ± SE**	**Jhansi ± SE**	**Shillongani ± SE**	**Jabalpur ± SE**	**Mean**
T_1_	*Trichoderma harzianum* T-6 (@10 g/kg seed)	196 ± 28.5	225 ± 5.8	313 ± 19.2	177 ± 9.1	227.75
T_2_	*Trichoderma harzianum* T-28 (@10 g/kg seed)	263 ± 50.5	209 ± 8.7	286 ± 12.0	181 ± 3.1	234.75
T_3_	*Trichoderma viride* T-18 (@10 g/kg seed)	292 ± 8.7	168 ± 19.2	297 ± 9.4	185 ± 8.7	235.50
T_4_	TrichoBARC (@10 g/kg seed)	291 ± 4.7	186 ± 6.6	288 ± 15.8	185 ± 9.3	237.50
T_5_	Propineb (Antracol) @3 g/kg seed	238 ± 66.7	203 ± 4.0	287 ± 12.6	178 ± 4.8	226.50
T_6_	Hexaconazol + Zineb (Avtar) @3 g/kg seed	301 ± 10.8	204 ± 17.1	286 ± 20.0	182 ± 5.8	243.25
T_7_	*Trichoderma harzianum* T-6 + Propineb (@10 g + 1.5 g/kg seed)	182 ± 2.6	273 ± 12.9	309 ± 5.7	182 ± 2.3	236.50
T_8_	Control	181 ± 13.9	140 ± 8.9	277 ± 15.1	175 ± 7.5	193.25
	C.D. at 5%	69.60	25.90	7.91	1.40	
	C.V. (%)	19.54	8.80	13.02	1.368	

**TABLE 2C:** Evaluation of new stains of *Trichoderma* along with fungicides for the management of collar rot of chickpea (2017–2018) – Yield (kg/ha).

**S. No.**	**Treatment Details**	**Raipur ± SE**	**Jhansi ± SE**	**Shillongani ± SE**	**Jabalpur ± SE**	**Mean**
T_1_	*Trichoderma harzianum* T-6 (@10 g/kg seed)	676 ± 96.6	564 ± 22.5	733 ± 7.3	520 ± 23.1	623.25
T_2_	*Trichoderma harzianum* T-28 (@10 g/kg seed)	902 ± 160.1	410 ± 40.0	848 ± 7.3	497 ± 3.8	664.25
T_3_	*Trichoderma viride* T-18 (@10 g/kg seed)	952 ± 44.6	226 ± 25.5	825 ± 8.7	603 ± 6.0	651.50
T_4_	TrichoBARC (@10 g/kg seed)	925 ± 61.2	282 ± 24.4	878 ± 6.0	647 ± 11.6	683.00
T_5_	Propineb (Antracol) @3 g/kg seed	995 ± 162.1	387 ± 28.3	800 ± 11.5	436 ± 6.6	654.50
T_6_	Hexaconazol + Zineb (Avtar) @3 g/kg seed	980 ± 8.3	324 ± 29.5	927 ± 10.1	494 ± 13.0	681.25
T_7_	*Trichoderma harzianum* T-6 + Propineb (@10 g + 1.5 g/kg seed)	796 ± 74.5	674 ± 31.3	977 ± 10.1	500 ± 15.5	736.75
T_8_	Control	584 ± 76.5	194 ± 3.7	624 ± 9.2	383 ± 64.2	446.25
	C.D. at 5%	223	62	7	75	
	C.V. (%)	17.82	10.99	0.58	8.32	

**TABLE 3A:** Evaluation of new stains of *Trichoderma* along with fungicides for the management of collar rot of chickpea (2018–2019) – Seedling emergence.

**S. No.**	**Treatment Details**	**Raipur ± SE**	**Jhansi ± SE**	**Shillongani ± SE**	**Jabalpur ± SE**	**Mean**
T_1_	*Trichoderma harzianum* T-6 (@10 g/kg seed)	219 ± 17.8	231 ± 5.3	330 ± 1.8	180 ± 3.1	240.00
T_2_	*Trichoderma harzianum* T-28 (@10 g/kg seed)	211 ± 25.9	204 ± 7.5	345 ± 2.3	184 ± 4.2	236.00
T_3_	*Trichoderma viride* T-18 (@10 g/kg seed)	173 ± 13.6	143 ± 6	344 ± 3.0	182 ± 2.9	210.50
T_4_	TrichoBARC (@10 g/kg seed)	286 ± 11.9	179 ± 1.2	349 ± 2.9	186 ± 0.3	250.00
T_5_	Propineb (Antracol) @3 g/kg seed	209 ± 17.9	207 ± 13.3	330 ± 2.9	186 ± 1.2	233.00
T_6_	Hexaconazol + Zineb (Avtar) @3 g/kg seed	232 ± 13.2	203 ± 4.5	353 ± 2.4	178 ± 1.2	241.50
T_7_	*Trichoderma harzianum* T-6 + Propineb (@10 g + 1.5 g/kg seed)	260 ± 14.4	288 ± 3.2	366 ± 3.1	180 ± 0.9	273.50
T_8_	Control	143 ± 10.7	130 ± 9.6	284 ± 2.9	170 ± 1.5	240.00
	C.D. at 5%	50.130	23.078	5.690	7.274	
	C.V. (%)	13.068	6.588	0.952	2.271	

**TABLE 3B:** Evaluation of new stains of *Trichoderma* along with fungicides for the management of collar rot of chickpea (2018–2019) – Disease incidence (%).

**S. No.**	**Treatment Details**	**Raipur ± SE**	**Jhansi ± SE**	**Shillongani ± SE**	**Jabalpur ± SE**	**Mean**
T_1_	*Trichoderma harzianum* T-6 (@10 g/kg seed)	22.82 ± 9.8 (28.459)	22.33 ± 2.2 (28.155)	31.80 ± 1.3 (34.30)	15.333 ± 2.73 (22.866)	23.07
T_2_	*Trichoderma harzianum* T-28 (@10 g/kg seed)	30.57 ± 11.8 (33.495)	32.00 ± 3.2 (34.402)	22.20 ± 0.7 (28.10)	18.867 ± 3.1 (25.578)	25.91
T_3_	*Trichoderma viride* T-18 (@10 g/kg seed)	34.04 ± 5.5 (35.536)	52.00 ± 5.2 (46.129)	24.20 ± 0.9 (29.50)	14.917 ± 0.7 (22.701)	31.29
T_4_	TrichoBARC (@10 g/kg seed)	14.39 ± 7.3 (22.199)	40.00 ± 4.0 (39.215)	16.80 ± 1.2 (24.20)	20.48 ± 2.5 (26.811)	22.92
T_5_	Propineb (Antracol) @3 g/kg seed	23.37 ± 8.7 (28.839)	30.67 ± 3.1 (33.486)	28.40 ± 1.6 (32.2)	20.333 ± 3.2 (26.644)	25.69
T_6_	Hexaconazol + Zineb (Avtar) @3 g/kg seed	25.00 ± 8.8 (29.938)	32.00 ± 3.2 (34.425)	12.90 ± 1.8 (20.90)	17.847 ± 3.5 (24.777)	21.94
T_7_	*Trichoderma harzianum* T-6 + Propineb (@10 g + 1.5 g/kg seed)	9.18 ± 4.6 (17.376)	4.00 ± 0.4 (11.277)	7.30 ± 0.4 (15.70)	31.667 ± 2.0 (34.209)	13.04
T_8_	Control	38.14 ± 9.5 (38.038)	56.67 ± 5.7 (48.830)	58.10 ± 1.7 (49.60)	29.667 ± 0.9 (32.984)	45.64
	C.D. at 5%	6.911	5.020	2.150	3.619	
	C.V. (%)	13.369	8.232	4.150	7.561	

**TABLE 3C:** Evaluation of new stains of *Trichoderma* along with fungicides for the management of collar rot of chickpea (2018–2019) – Yield (kg/ha).

**S. No.**	**Treatment Details**	**Raipur ± SE**	**Jhansi ± SE**	**Shillongani ± SE**	**Jabalpur ± SE**	**Mean**
T_1_	*Trichoderma harzianum* T-6 (@10 g/kg seed)	570.00 ± 26.5	567 ± 13.8	765 ± 2.08	481 ± 12.1	595.75
T_2_	*Trichoderma harzianum* T-28 (@10 g/kg seed)	693.33 ± 30.9	426 ± 12.6	890 ± 30.6	525 ± 46.7	633.58
T_3_	*Trichoderma viride* T-18 (@10 g/kg seed)	673.33 ± 23.1	222 ± 11.3	860 ± 23.1	581 ± 42.2	584.08
T_4_	TrichoBARC (@10 g/kg seed)	836.67 ± 28.4	262 ± 15.4	935 ± 15.3	500 ± 12.0	633.42
T_5_	Propineb (Antracol) @3 g/kg seed	616.33 ± 23.6	409 ± 5.2	830 ± 20.8	596 ± 52.3	612.83
T_6_	Hexaconazol + Zineb (Avtar) @3 g/kg seed	735.00 ± 17.3	314 ± 5.2	980 ± 20.8	530 ± 45.0	639.75
T_7_	*Trichoderma harzianum* T-6 + Propineb (@10 g + 1.5 g/kg seed)	773.33 ± 20.3	671 ± 17.4	1030 ± 20.8	442 ± 29.8	729.08
T_8_	Control	438.33 ± 28.9	196 ± 9.3	650 ± 36.1	360 ± 6.4	411.08
	C.D. at 5%	71.861	29.246	78.3	70.87	
	C.V. (%)	6.097	4.308	5.1	7.978	

### The Formulation Enhanced Chickpea Yield in “On-Farm” Demonstration Trials

**FIGURE 12:**
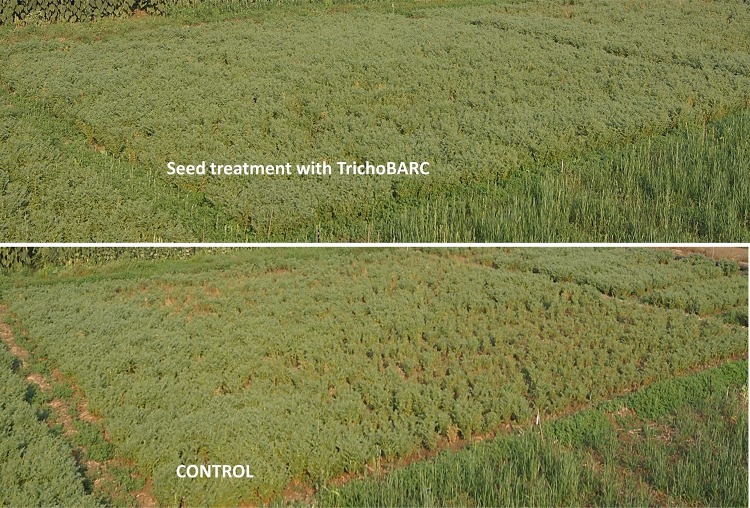
Demonstration trial on control of collar rot of chickpea in experimental field at Raipur (2015–2016).

We demonstrated the potential of the formulation to enhance chickpea yield at Raipur, Chhattisgarh (for chickpea) over 2 years. In the 2015–2016 demonstration trial in Raipur, TrichoBARC-treated chickpea plot (112 m^2^) recorded 54.2 kg bundle weight (biomass production) and 14 kg grain yield, compared to 29.16 and 8.8 kg, respectively, in the control plot; the TrichoBARC-treated plot had lower incidence of seedling mortality (Figure 12). In 2016–2017, in a bigger plot size (1512 m^2^), the grain yield was 161 kg in the TrichoBARC-treated plot, compared to 142 kg in control, and bundle weight was 386 and 271 kg per plot, respectively.

### TrichoBARC Seed Treatment Improved Lentil Yield in Farmers’ Field

**FIGURE 13:**
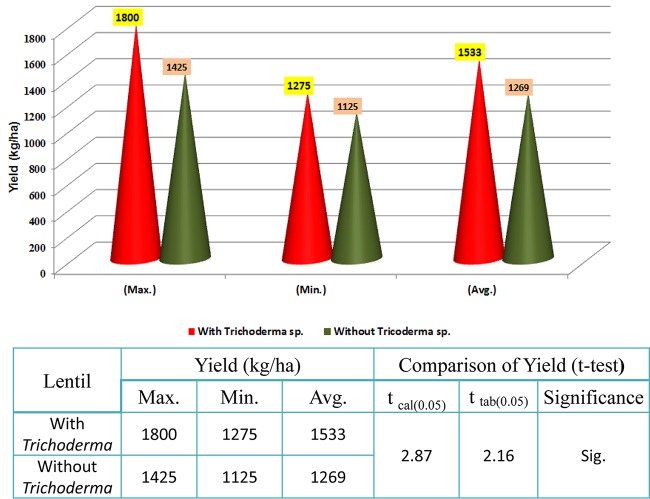
Yield data of farmers’ field trials taken at 30 farmers’ fields in Nadia district of West Bengal (2017–2018).

**FIGURE 14:**
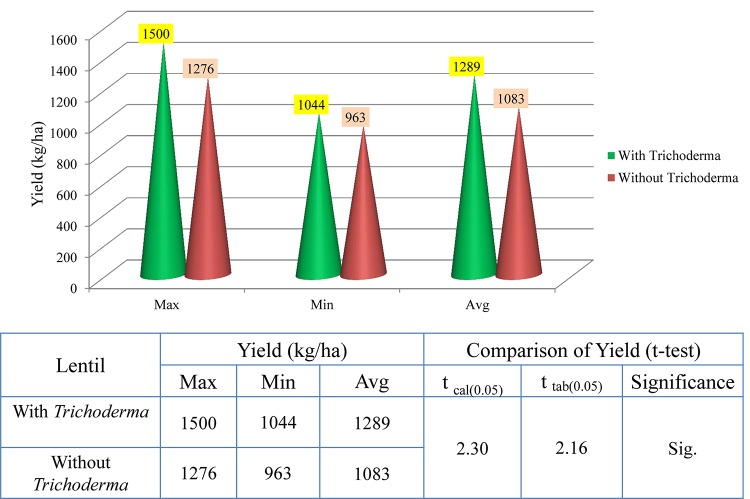
Yield data of farmers’ field trials taken at 30 farmers’ fields in Nadia district of West Bengal (2018–2019).

In 2017–2018, trials were taken up in 30 lentil farmers’ field in West Bengal. On average, yield per hectare was 1,533 kg, compared to 1,269 in the non-treated field, with the maximum yield recorded in the TrichoBARC-treated field being 1,800 kg/ha and the minimum being 1,275 kg/ha, compared to 1425 and 1125 kg/ha in control fields, and the yield gain was statistically significant (Figure 13). In 2018–2019 too, average yield gain in 30 farmers’ fields across four villages in Nadia district of West Bengal was more than 200 kg/ha (Figure 14).

### The Formulation Developed Is Non-toxic to Mammals, Birds, and Fish

The toxicological studies of TrichoBARC formulation on rat, mice, rabbit, chicken, pigeon, and a freshwater fish *D. rerio* revealed that the formulation is non-toxic to these test animals (Table 4).

**TABLE 4:** Toxicity of TrichoBARC formulation on mammals, birds, and fish.

**S. No.**	**Study Title**	**Results**
1	Acute Oral Toxicity/Pathogenicity in Wistar Rat	No toxicity, infectivity, or pathogenicity
2	Acute Oral Toxicity/Pathogenicity in CD 1 Mice	No toxicity, infectivity, or pathogenicity
3	Acute Pulmonary Toxicity/Pathogenicity Study in Wistar Rat	No toxicity, infectivity, or pathogenicity
4	Acute Dermal Toxicity/Pathogenicity Study in New Zealand White Rabbits	No treatment-related toxicity, pathogenicity, or skin irritation
5	Acute Intraperitoneal Toxicity/Pathogenicity Study in Wistar Rats	No treatment-related toxicity, infectivity, or pathological effects
6	Acute Dermal Irritation in New Zealand White Rabbits	Non-irritating
7	Acute Eye Irritation in New Zealand White Rabbits	Non-irritating
8	Acute Oral Toxicity Study in Chicken	Non-toxic and non-virulent
9	Acute Oral Toxicity Study in Pigeon	Non-toxic and non-virulent
10	Acute Toxicity Study to Freshwater Fish, *Danio rerio*	>100 mg/L (non-toxic and non-virulent)

## Summary and Conclusion

*Trichoderma* spp. are widely used in agriculture, several formulations being available as biofungicides and plant growth promoters. *T. virens* is also commercially sold as a biofungicide^1^ and plant growth promoter (in combination with *Bacillus amyloliquefaciens*^2^) in the United States. Even though natural strains of *Trichoderma* are effective, there is always a scope for strain improvement. Induced mutation is a powerful tool for genetic enhancement of microbes. For example, the industrial strain RutC 30 of *Trichoderma reesei* (used for enzyme production) is derived from a natural strain QM6A through repeated mutagenesis resulting in 15- to 20-fold improvement in cellulase biosynthesis (Peterson and Nevalainen, 2012). Another example is the enhancement of penicillin biosynthesis – it has been possible to enhance the titer by 85-fold through mutation (Ziemons et al., 2017). There are several such examples in industry, but only a few in microbes of agricultural importance. Even though attempts were made to “improve” biocontrol potential of *Trichoderma* strains through mutagenesis, seldom they have been commercialized. Biocontrol potential of *Trichoderma* spp. can also be enhanced by improving formulation strategy. In the present study, we have demonstrated that it is possible to improve the antagonistic and biocontrol potential of a strain of *T. virens* that has widely been studied for biocontrol properties as well as genetics. We have combined genetic enhancement with novel formulation strategy, and through extensive evaluation over many years in several locations, we demonstrate the commercial potential of such a novel formulation. The use of tamarind seeds as a mass multiplication medium has unique advantages. In developing countries, including India, food grains like sorghum are extensively used for growing *Trichoderma* at industry scale, while tamarind seeds are by-products of tamarind pulp that are very popular as a culinary ingredient throughout India. This technology thus replaces expensive food grains with cheap tamarind seeds. Moreover, tamarind seed-based formulations, being sticky in nature, do not require addition of stickers/spreaders like carboxy-methyl cellulose and detergents that are routinely used in *Trichoderma* formulations. This formulation described here is thus an organic formulation *sensu stricto*. The extensive field testing data (including farmers’ field) that we present here clearly establish our formulation as an excellent technology for crop production, especially pulses. The formulation has been proven to not only be effective in controlling collar rot, a serious problem in more than 500 crop species, but also cause robust plant growth (Supplementary Figures S1, S2). We also noticed early flowering (by 7–10 days) in TrichoBARC-treated chickpea and lentil under field conditions at multiple locations. We are yet to study the mechanism of improved biocontrol and growth enhancement, which could be due to the overproduction of secondary metabolites, and constitutive overexpression of many genes known to be involved in mycoparasitism (like chitinases, chitosanases, and glucanases), root colonization (like pectinases), and SSCPs (known to play role as elicitors and effectors). The formulation developed by us using a novel mutant combined with a novel formulation strategy thus holds promise for commercial success as a novel plant bio-inoculant. Additionally, through toxicology studies, we also provide evidence that this formulation is safe, which adds to the commercial prospect of this technology described here.

## Data Availability

The datasets generated for this study can be found in the RNAseq data included as Supplementary File.

## Ethics Statement

This study was performed in accordance with the USEPA, OPPTS microbial pesticide test guidelines, and Committee for the Purpose of Control and Supervision of Experiments on Animals (CPCSEA), Animal Welfare Division of Ministry of Environment, Forest and Climate Change, Government of India guidelines under the Prevention of Cruelty to Animals Act 1960 (formed in 1964 and revived in 1998). The experiments were approved and monitored by the Institutional Animal Ethics Committee (IAEC), IIBAT.

## Author Contributions

PM, SM, PS, and GM performed the laboratory and greenhouse experiments. PM and PS prepared the formulations for field studies. AG, AK, NK, PR, KS, RN, AT, SB, MA, DP, AW, RT, and DS performed the field trials. PM conceptualized, designed, and coordinated the studies, and wrote the manuscript.

## Conflict of Interest Statement

The authors declare that the research was conducted in the absence of any commercial or financial relationships that could be construed as a potential conflict of interest.

## Footnotes

1 http://certisusa.com/pest_management_products/biofungicides/soilgard_12g_microbial_fungicide.htm

2 https://www.aganytime.com/dekalb/protection/Pages/Monsanto-BioAg.aspx
