# Supplementary material for: A Novel Seed-Dressing Formulation Based on an Improved Mutant Strain of Trichoderma virens, and Its Field Evaluation
Source: Front Microbiol. 2019 Aug 30;10:1910. doi: 10.3389/fmicb.2019.01910 (PMC6730527; doi:10.3389/fmicb.2019.01910)
Supplement: Supplementary file 2 [file Presentation_1.PPTX]

## Slide 1
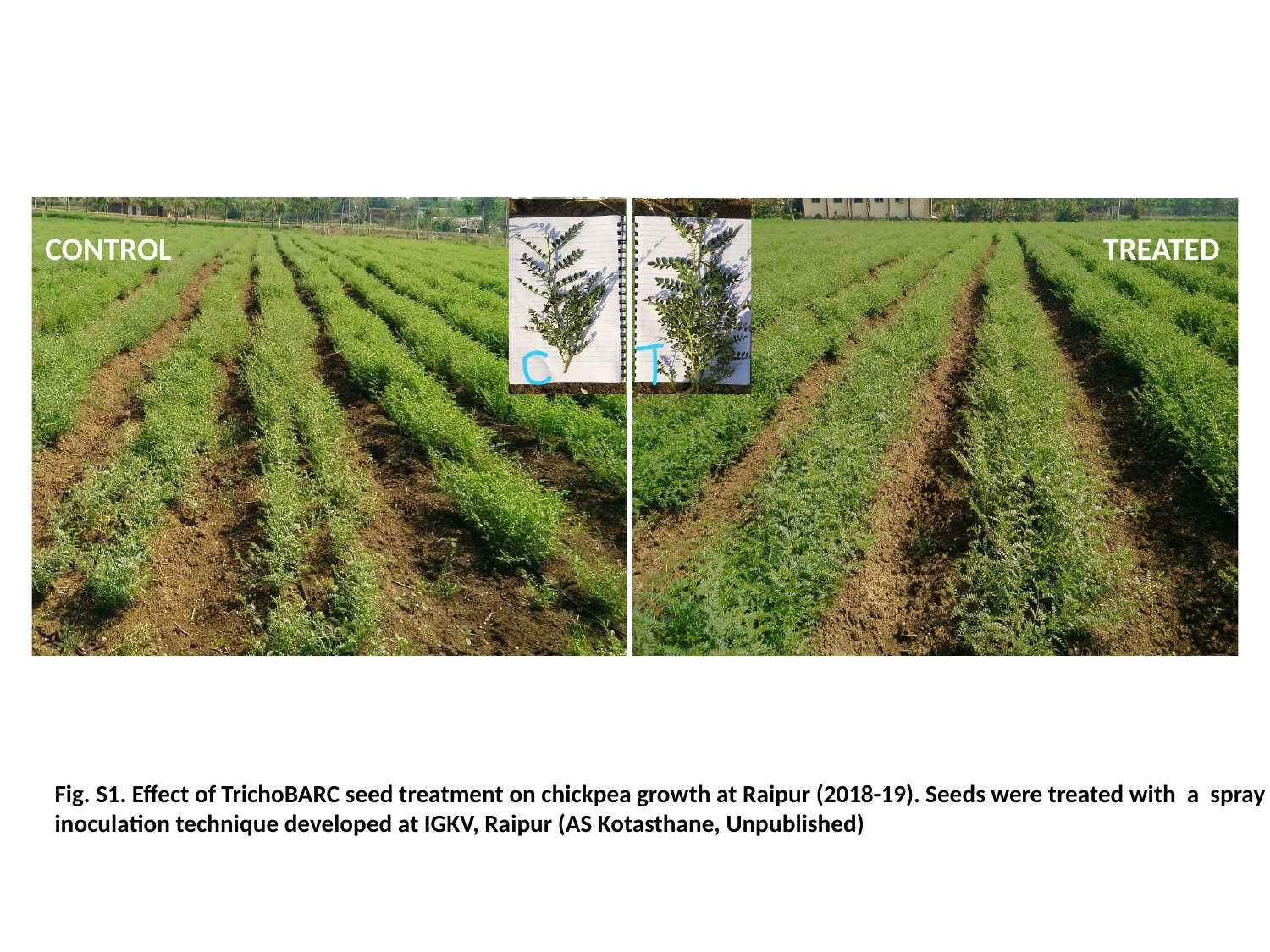

CONTROL
TREATED
Fig. S1. Effect of TrichoBARC seed treatment on chickpea growth at Raipur (2018-19). Seeds were treated with a spray
inoculation technique developed at IGKV, Raipur (AS Kotasthane, Unpublished)

## Slide 2
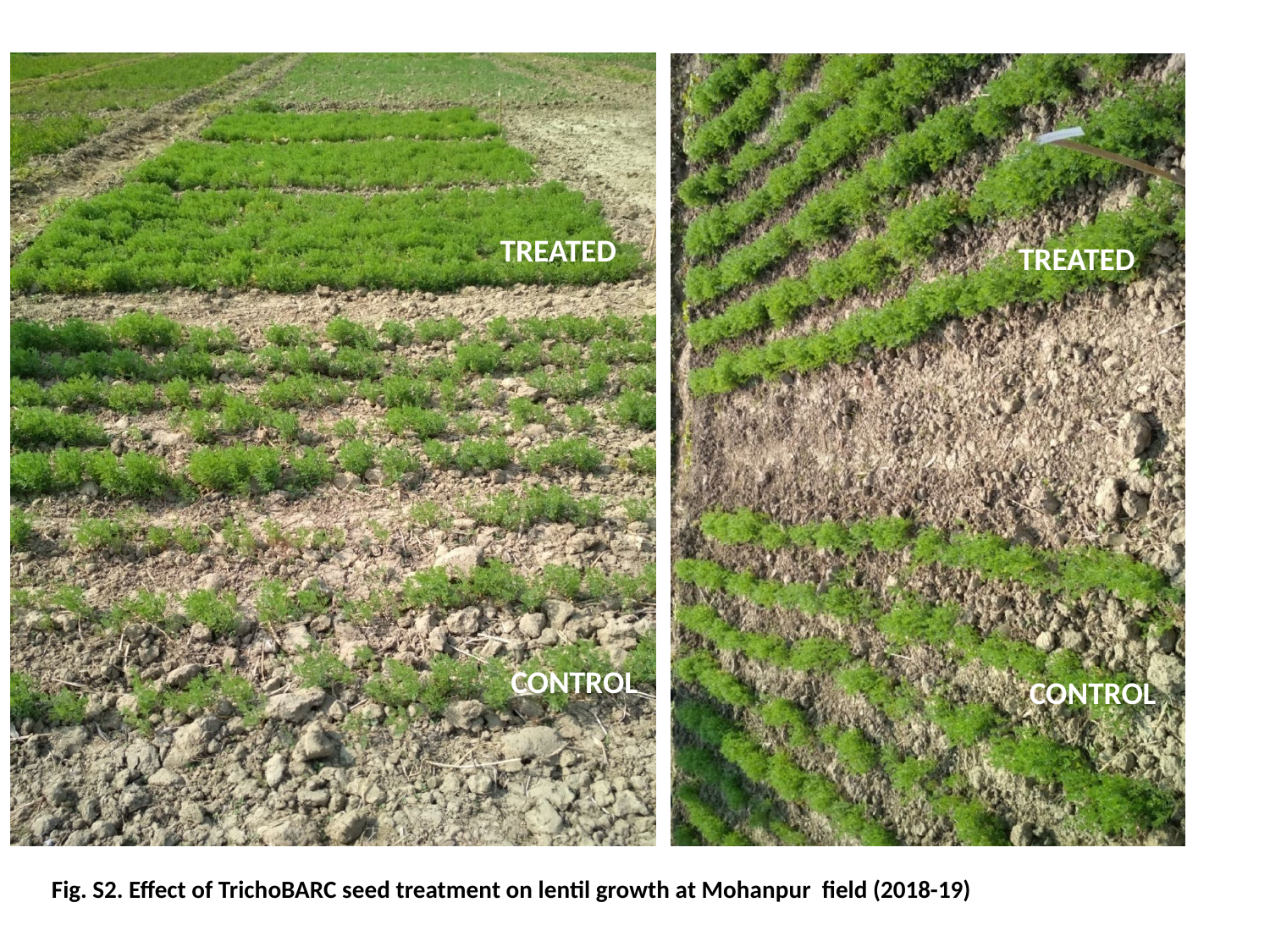

TREATED
TREATED
CONTROL
CONTROL
Fig. S2. Effect of TrichoBARC seed treatment on lentil growth at Mohanpur field (2018-19)
